# Transcriptional responses of *Neisseria gonorrhoeae* to glucose and lactate: implications for resistance to oxidative damage and biofilm formation

**DOI:** 10.1128/mbio.01761-24

**Published:** 2024-07-16

**Authors:** Julio C. Ayala, Jacqueline T. Balthazar, William M. Shafer

**Affiliations:** 1 Department of Microbiology and Immunology, Emory University School of Medicine, Atlanta, Georgia, USA; 2 Division of STD Prevention, National Center for HIV, Viral Hepatitis, STD, and TB Prevention, Centers for Disease Control and Prevention, Atlanta, Georgia, USA; 3 Emory Antibiotic Resistance Center, Emory University School of Medicine, Atlanta, Georgia, USA; 4 Laboratories of Bacterial Pathogenesis, Veterans Affairs Medical Center, Decatur, Georgia, USA; Harvard Medical School, Boston, Massachusetts, USA

**Keywords:** *Neisseria gonorrhoeae*, iron transport, lactate utilization, H_2_O_2 _resistance

## Abstract

Understanding how bacteria adapt to different environmental conditions is crucial for advancing knowledge regarding pathogenic mechanisms that operate during infection as well as efforts to develop new therapeutic strategies to cure or prevent infections. Here, we investigated the transcriptional response of *Neisseria gonorrhoeae*, the causative agent of gonorrhea, to L-lactate and glucose, two important carbon sources found in the host environment. Our study revealed extensive transcriptional changes that gonococci make in response to L-lactate, with 37% of the gonococcal transcriptome being regulated, compared to only 9% by glucose. We found that L-lactate induces a transcriptional program that would negatively impact iron transport, potentially limiting the availability of labile iron, which would be important in the face of the multiple hydrogen peroxide attacks encountered by gonococci during its lifecycle. Furthermore, we found that L-lactate-mediated transcriptional response promoted aerobic respiration and dispersal of biofilms, contrasting with an anaerobic condition previously reported to favor biofilm formation. Our findings suggest an intricate interplay between carbon metabolism, iron homeostasis, biofilm formation, and stress response in *N. gonorrhoeae*, providing insights into its pathogenesis and identifying potential therapeutic targets.

**IMPORTANCE:**

Gonorrhea is a prevalent sexually transmitted infection caused by the human pathogen *Neisseria gonorrhoeae*, with ca. 82 million cases reported worldwide annually. The rise of antibiotic resistance in *N. gonorrhoeae* poses a significant public health threat, highlighting the urgent need for alternative treatment strategies. By elucidating how *N. gonorrhoeae* responds to host-derived carbon sources such as L-lactate and glucose, this study offers insights into the metabolic adaptations crucial for bacterial survival and virulence during infection. Understanding these adaptations provides a foundation for developing novel therapeutic approaches targeting bacterial metabolism, iron homeostasis, and virulence gene expression. Moreover, the findings reported herein regarding biofilm formation and L-lactate transport and metabolism contribute to our understanding of *N. gonorrhoeae* pathogenesis, offering potential avenues for preventing and treating gonorrhea infections.

## INTRODUCTION

Gonorrhea is one of the oldest sexually transmitted infections (STIs) known to humans with records of its symptoms described as far back as the 14th century (1). *Neisseria gonorrhoeae* (Ng) is the etiologic agent of gonorrhea and caused 648,056 reported cases in the USA in 2022 (2) and an estimated 82 million worldwide each year (3). Contributing to the high global burden of the disease are the lack of a preventive vaccine (4) and the emergence of gonococcal strains that resist previous or current front-line antibiotics including the last remaining antibiotic for empiric monotherapy (ceftriaxone) in the USA and elsewhere [reviewed in references (5, 6)].

As a host-restricted pathogen, *N. gonorrhoeae* has developed unique mechanisms to persist during infection in human genital and at extra-genital sites such as the rectal and pharyngeal mucosa (7). Although numerous advances have been made regarding the mechanisms by which gonococci attach and invade epithelial cells as well as their capacity to resist mediators of innate host defenses [reviewed in references (8–10)] much remains to be learned as to how this human pathogen responds to environmental conditions that it would encounter during infection. In this regard, several studies have determined transcriptional regulatory systems that modulate the expression of genes in response to certain stress conditions (e.g., iron limitation, oxidative stress, oxygen deprivation, or interaction with neutrophils) (11–17). An important, but under-studied physiologic condition likely to be important during gonococcal infection is how the colonizing bacteria respond to the availability of carbon sources required for their growth. Recently, we described the importance of the lactate permease (LctP) for Ng resistance to hydrogen peroxide and showed that the expression of *lctP* is negatively controlled by the GntR-type regulator GdhR (18). However, the molecular basis for resistance to H_2_O_2_ killing provided by LctP expression and the consequent L-lactate transport and metabolism is not well understood. LctP is an important gonococcal virulence factor required for colonization of the lower genital tract of female mice and for determining in some strains resistance to killing by normal human serum (19, 20). Based on these findings, we examined the transcriptional response made by Ng during growth when L-lactate or glucose are the main carbon sources. Our findings indicate unique transcriptional patterns made by Ng in response to L-lactate and that the products of regulated genes influence systems proposed to be critical for pathogenesis.

## RESULTS

### Genome-wide transcriptional responses of gonococci to L-lactate and glucose in the culture medium

**Fig 1:**
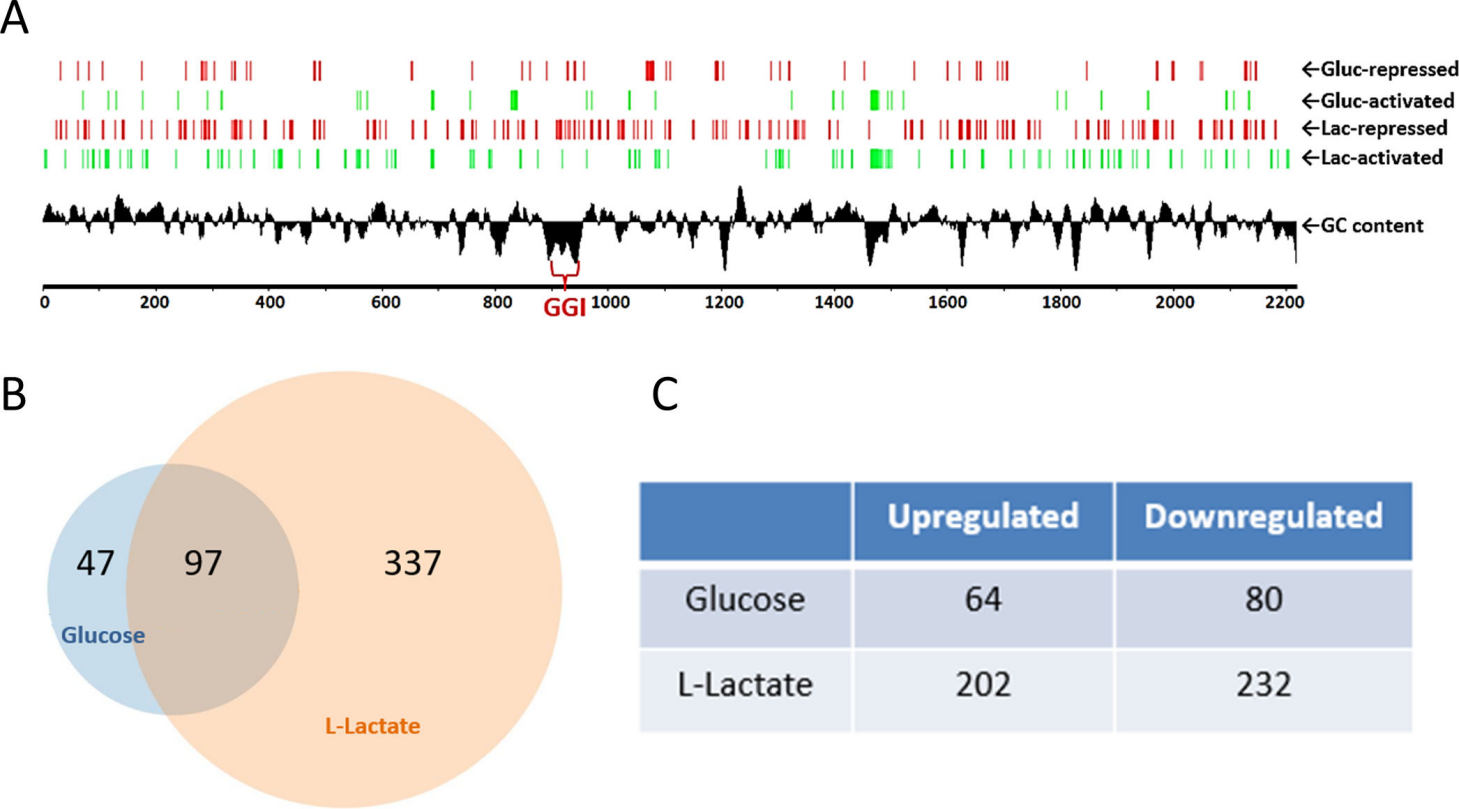
Genome-wide transcriptional landscape changes of *N. gonorrhoeae* to L-lactate and glucose in the culture medium. (**A**) Graphic representation of gonococcal genes activated (green) and repressed (red) by glucose and L-lactate determined by RNA-Seq. *N. gonorrhoeae* cells were grown in GC broth at 1 or 10 mM of each sugar and collected at the mid-logarithmic phase for RNA-Seq. The numbering indicates the chromosome coordinates in kb pairs. The black plot is the deviation from the average GC content of the genome. GGI: gonococcal genetic island. (**B**) Venn diagram of glucose and L-lactate co-regulated genes (at fold-change ≥2). (**C**) The number of glucose and L-lactate up- and down-regulated genes (at fold-change ≥2).

**Fig 2:**
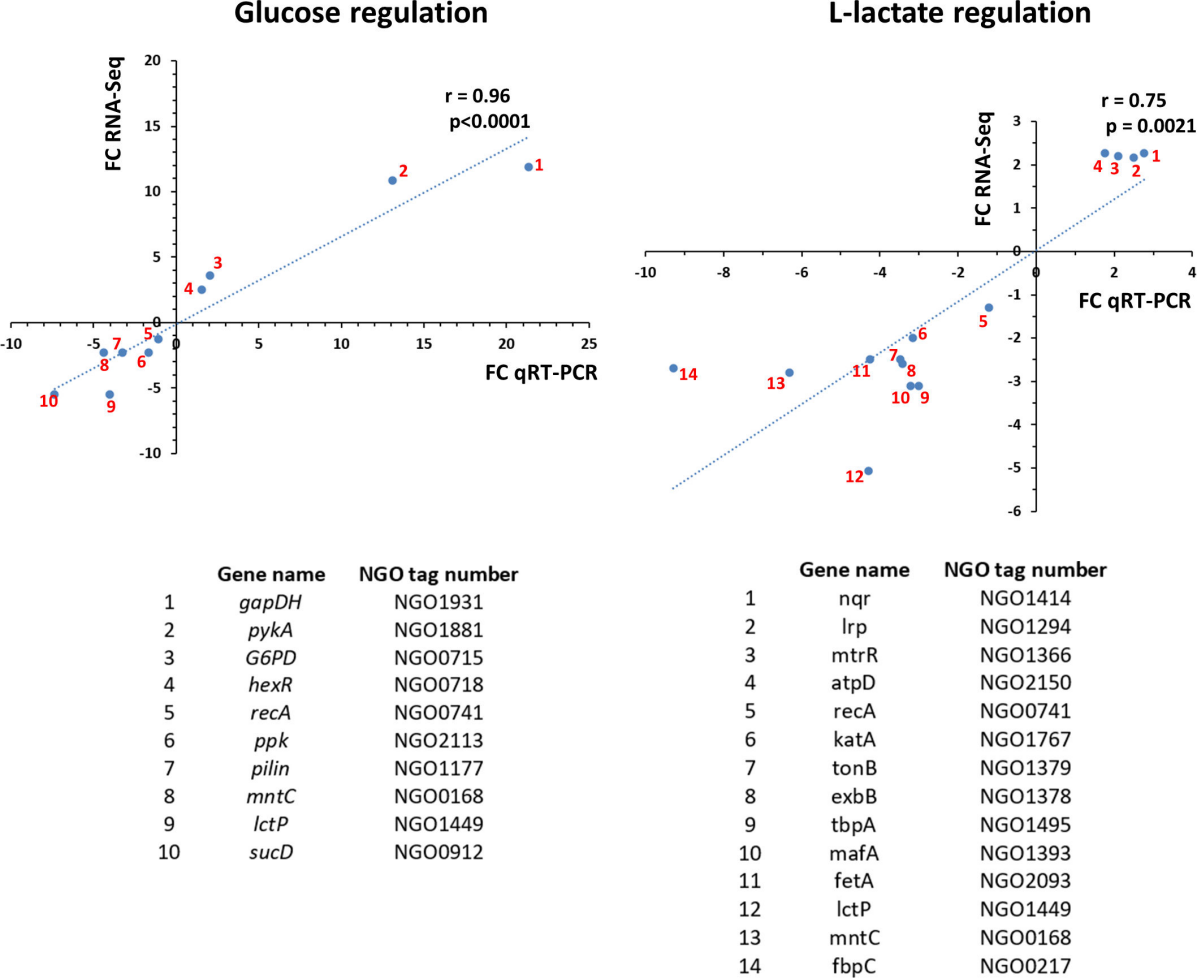
Validation of the RNA-Seq study using qRT-PCR in a subset of 10 and 14 genes for the glucose and L-lactate regulon, respectively. A subset of statistically significant up- or down-regulated genes (except for *recA*, which is housekeeping gene not regulated) for the observed glucose and L-lactate regulons were selected for qRT-PCR validation. *N. gonorrhoeae* FA19 was grown in GC-broth containing 1 or 10 mM of glucose with a constant concentration of 3 mM L-lactate, or 1 or 10 mM of L-lactate with a constant concentration of 1.5 mM glucose. Cells were collected at the mid-logarithmic phase for RNA extraction. Each gene point is scattered based on the fold-change (FC) regulation between 1 and 10 mM of each sugar. The nonparametric Spearman correlation coefficients (R) and *P* values (two-tailed) are indicated for the glucose (left) and L-lactate (right) transcriptomic analyses.

For the transcriptional profiling studies, performed by RNA-Seq, we tested concentrations of glucose or L-lactate in GC-broth that rendered similar initial growth rates (i.e., early to mid-logarithmic phase) using Ng strain FA19 *rpsL* K43R. For the glucose RNA-Seq study, we selected two concentrations (1 and 10 mM) with a fixed concentration of 3 mM L-lactate in both, and for the determination of the L-lactate regulon, we selected 1 and 10 mM of L-lactate with a fixed concentration of 1.5 mM glucose (Fig. S1). This approach resulted in similar growth curves up to the mid-exponential phase, where we collected samples for RNA extraction. We considered other strategies such as growing Ng in regular GC broth (containing 22 mM glucose) and then adding (or not) L-lactate but adding lactate to a medium containing glucose increases Ng growth rate (21). This would have resulted in samples situated at different points in the growth curve; thus, RNA-seq data would have been confounded by different growth states and not only reflected responses to lactate/glucose. The RNA-Seq bioinformatic analysis revealed that L-lactate regulates 988 genes (434 at ≥two-fold change regulation) while glucose regulates 227 genes (144 at ≥two-fold change regulation) (Fig. 1; Tables S1 and S2). In all, 97 genes were co-regulated by glucose and L-lactate (Fig. 1B). We note that a sub-set of co-regulated genes might be solely regulated by glucose since L-lactate is a gluconeogenic carbohydrate (22). Evidence for sole regulation by glucose is that the RNA-Seq analysis found that both glucose and L-lactate repressed the expression of *lctP* (Tables S1 and S2) at 10 mM compared to 1 mM. However, glucose, but not L-lactate, when present at physiological concentrations [i.e., up to 5 mM in women’s vaginal secretions (23)], was able to repress *lctP* expression (18). This observation together with the fact that neither glucose nor L-lactate allosterically regulates the *lctP* repressor GdhR (18) suggests that only when L-lactate accumulates at a high concentration can the cellular glucose pool *via* gluconeogenesis be replenished and thus exert repression on *lctP*. In this regard, carbon catabolite repression of the utilization of secondary carbohydrates to glucose in Ng does not seem to be connected to a glucose transport phosphotransferase system (PTS), but rather to the internal pool of glucose and the resulting energetic state of the cells (18). To validate the results of the RNA-Seq bioinformatic analysis, we selected subsets of 9 and 13 genes up- or down-regulated by glucose and L-lactate, respectively, to conduct quantitative reverse transcription-PCR (qRT-PCR) experiments (Fig. 2). The results showed significant correlation in gene regulation between RNA-Seq and qRT-PCR analyses for both carbohydrates.

**Fig 3:**
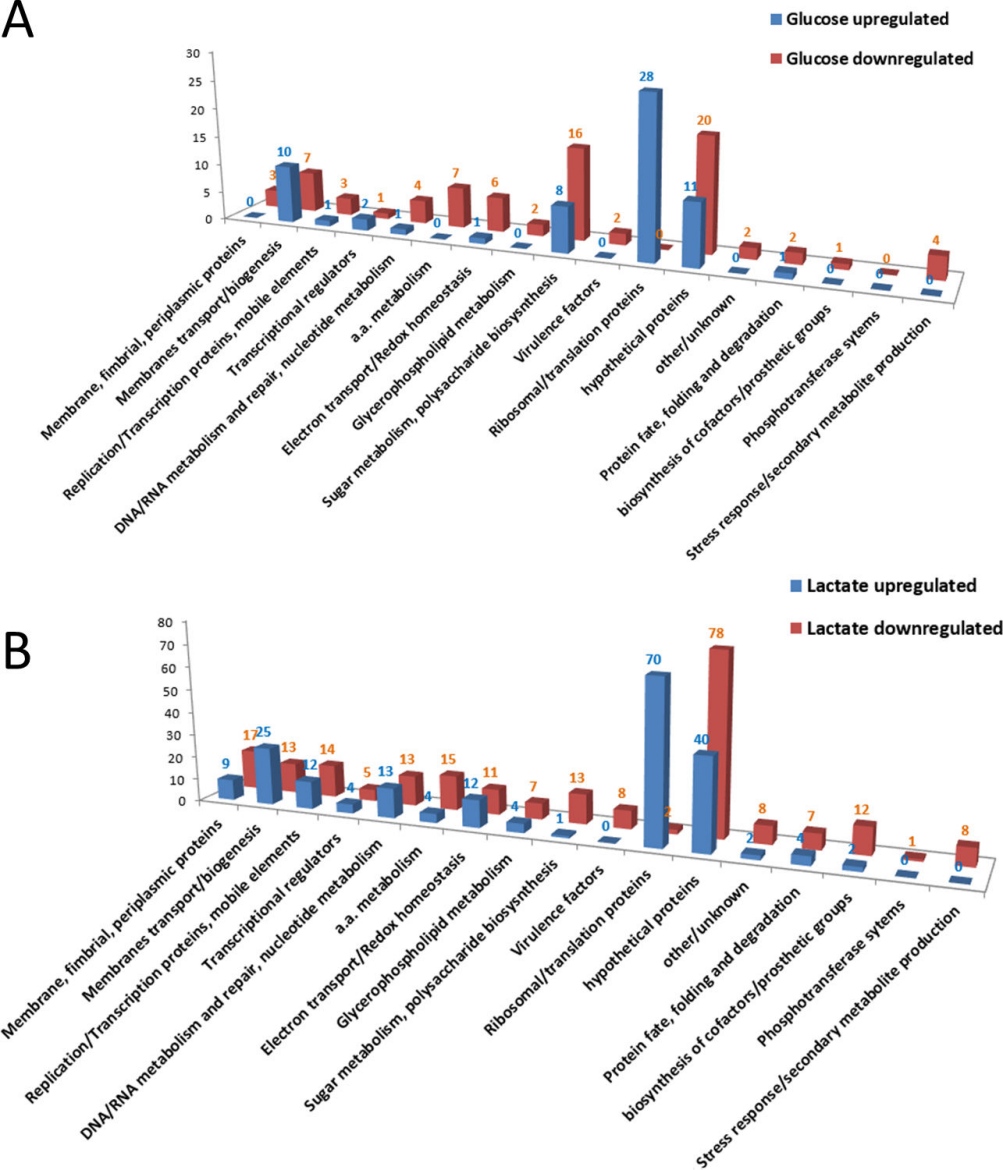
Representation of up- and down-regulated genes within the glucose (**A**) and L-lactate (**B**) RNA-seq-determined regulons grouped by functional categories. Shown on the *y*-axis is the number of genes within a functional category (*x*-axis) that were regulated ≥ two-fold.

Similarities among both regulons lie in that most up-regulated genes by both glucose and L-lactate are involved in translation (e.g., ribosomal protein genes), which is a high energy-consuming cellular process (Fig. 3). The majority of down-regulated genes by both sugars are hypothetical protein-encoding genes. Individually, glucose enhances the expression of glycolytic pathway genes [only the Entner-Doudoroff and the Pentose Phosphate glycolytic pathways are functional in Ng, while the Embden-Meyerhof-Parnas pathway is not (24)]. However, glucose represses TCA cycle-associated genes (Fig. 2; Table S2). This gene regulatory behavior by glucose has been shown before with the observation that acetate accumulates during glucose catabolism and is not further metabolized until glucose catabolism ceases; subsequently, acetate is oxidized by the TCA cycle (25). Furthermore, L-lactate enhances the expression of genes encoding respiratory enzymes and ATP synthase subunits (Fig. 2; Table S1). In this regard, L-lactate derived from cell-free supernatants of phagocytes and human serum was shown to increase the rate of oxygen consumption and metabolism of *N. gonorrhoeae* (26).

### Correlation of the L-lactate regulon with the hydrogen peroxide-exposure and iron regulons

**Fig 4:**
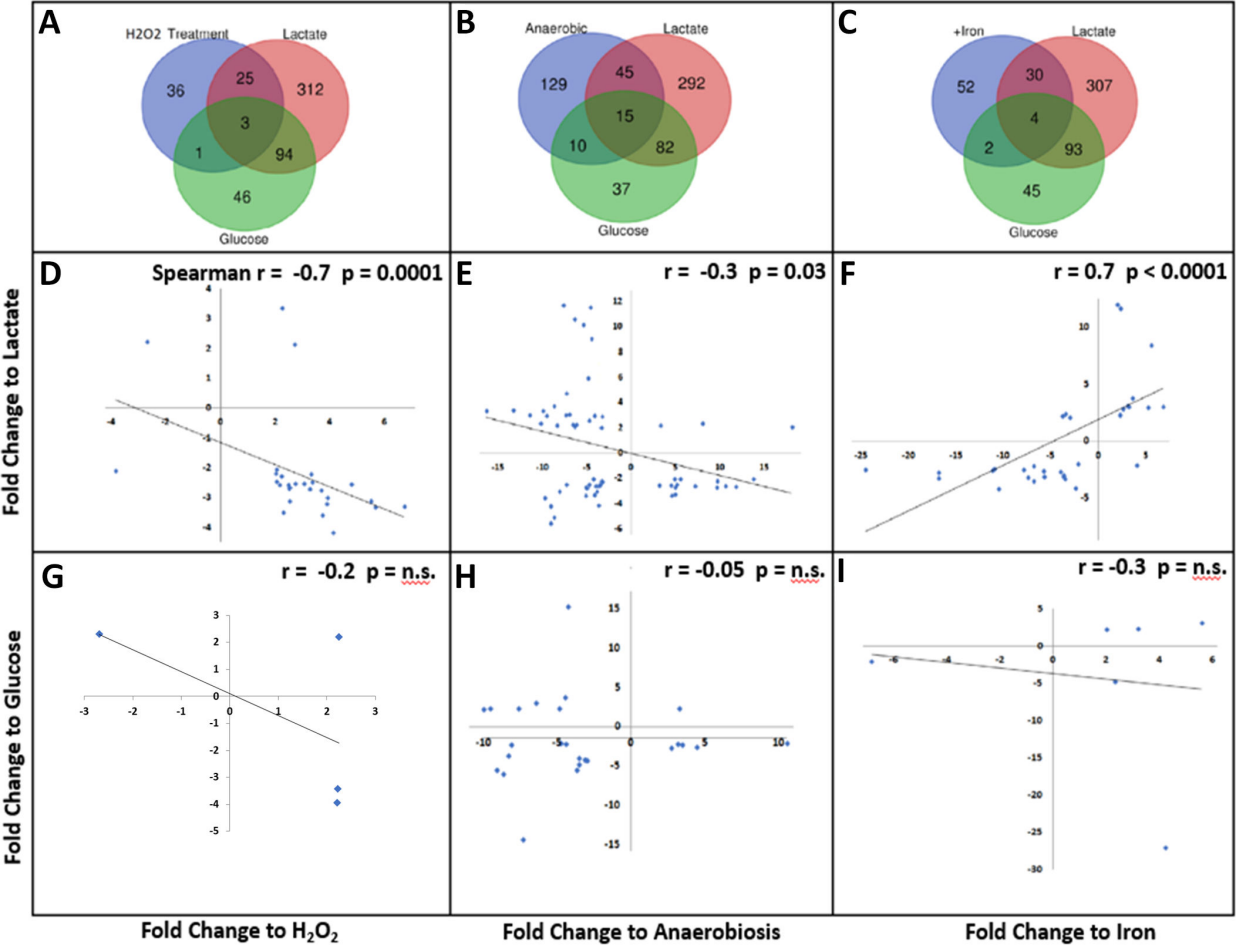
Correlation analysis of the L-lactate and glucose RNA-Seq-determined regulons with the regulon of different conditions faced by the gonococci during infection. Venn diagrams were drawn depicting the relationship of the L-lactate and glucose regulons with previously determined transcriptomes for sub-lethal hydrogen peroxide exposure [using the bioinformatic analysis compiled by (28) from original data reported by (14)] (**A**), anaerobic conditions (13) (**B**) and iron-replete conditions [using bioinformatic analysis compiled by (28) from original data reported by (11)] (**C**). Co-regulated genes between the L-lactate regulon and each of the three previously stated experimental conditions in a, b, and c were used to construct scatter plot graphs using the fold-change regulation as x,y variables and for correlation analysis (**D,E,F**). Correlation analysis between the glucose regulon and the regulon of the three experimental conditions are presented in (**G,H,I**). The nonparametric Spearman coefficient (R) and *P* values are indicated for each correlation analysis.

**Fig 5:**
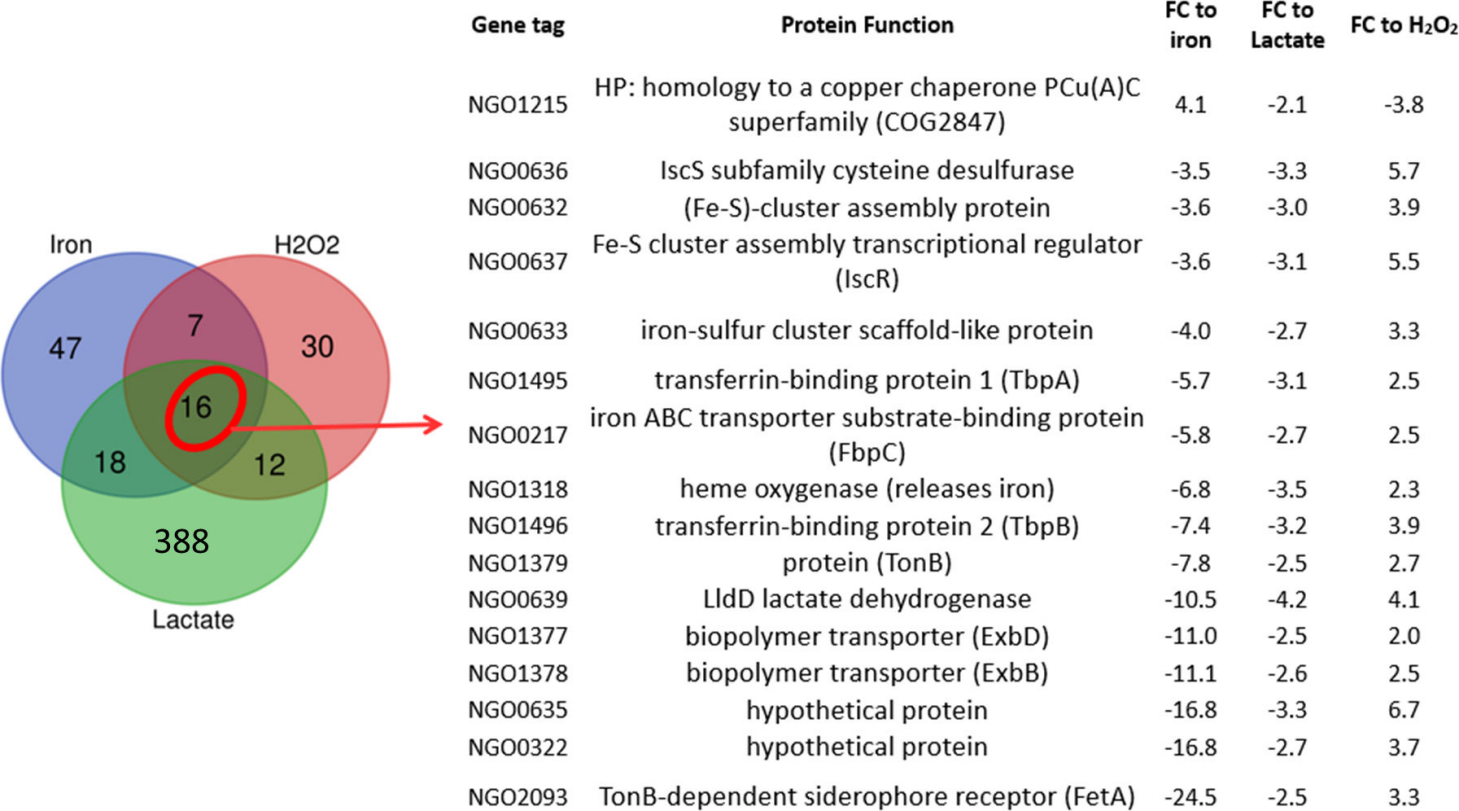
The subset of co-regulated genes between the L-lactate, iron-replete conditions, and sub-lethal hydrogen peroxide exposure regulons contains mainly iron transport and iron-sulfur cluster repair genes. FC: fold-change. Please see the legend in Fig. 4 for the source of data for the regulons described in the figure.

Inspection of the L-lactate regulon revealed a significant amount of repressed iron transport genes compared to the glucose regulon, suggesting that the transcriptomic rearrangement upon exposure to L-lactate encodes a genetic program aimed at reducing iron import (Table S3). Labile iron, that is, the pool of chelatable and redox-active iron, is particularly deleterious to bacterial cells in the presence of H_2_O_2_ because of its capacity to catalyze the Fenton reaction generating dangerous hydroxyl radicals (27). Thus, this specific change in the gene expression pattern in response to L-lactate transport and metabolism may explain, in part, the LctP-mediated protection from H_2_O_2_ killing that we have observed before (18). Accordingly, we compared the L-lactate transcriptome with previously determined transcriptomes from Ng exposed to sub-lethal levels of H_2_O_2_ (H_2_O_2_ regulon) (14) and to iron-replete conditions (11) (Fig. 4). A comparison of the L-lactate and the H_2_O_2_ regulons revealed a subset of 28 commonly regulated genes (Fig. 4a), whose regulations are significantly correlated in an inverse manner (Fig. 4d). This subset is mostly genes that are up-regulated by H_2_O_2_ while down-regulated by L-lactate. The same comparison showed that this co-regulation is specific for L-lactate since there is no correlation between the glucose and the H_2_O_2_ regulons (Fig. 4g). Similarly, comparison with the iron-replete regulon showed a significant direct correlation with the L-lactate regulon, but no correlation with the glucose regulon (Fig. 4f and i). Upon observation of the subset of co-regulated genes among the iron-replete, H_2_O_2_, and L-lactate regulons we next identified iron transport and iron-sulfur clusters repair genes (Fig. 5). The average fold-change in L-lactate regulation of iron-regulated genes was −3.1.

### L-lactate impacts Ng biofilm formation

**Fig 6:**
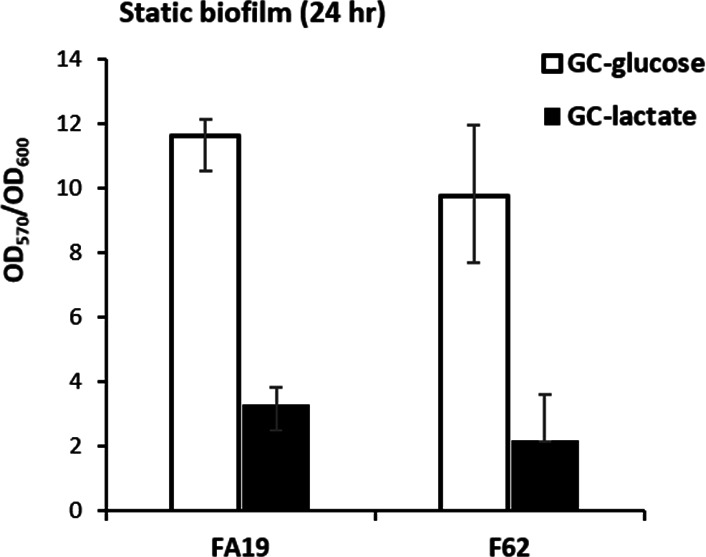
Effect of L-lactate on gonococcal biofilms. *Neisseria gonorrhoeae* strains FA19 and F62 were grown in GC broth containing either glucose (22 mM) or L-lactate (22 mM) in 96-well plates. Biofilm formation was quantified using the crystal violet staining method. The data are represented as the median (bar) plus/minus the 75% and 25% percentiles, respectively (error bars) of 4 biological replicates for FA19 and 3 for F62. For both strains, the difference in biofilm level in glucose vs L-lactate-grown gonococci was statistically significant using a nonparametric Mann-Whitney U-test (one-tailed *P* = 0.0143 for FA19 and *P* = 0.05 for F62).

Analysis of the L-lactate regulon showed that several nuclease-encoding genes are up-regulated in the presence of L-lactate, while several adhesin-encoding genes are down-regulated (Table 1). The gonococcal biofilm matrix has been shown to contain extracellular DNA (eDNA) and its incorporation into the biofilm is regulated by an extracellular nuclease (29). This nuclease called Nuc (NGO0969) and previously shown enzymes with biofilm-modifying activity such as the Type III methyltransferase M.NgoAX (NGO0545) (30), the peptidoglycan recycling NagZ (NGO0135) (31), or the DNA repair proteins MutL (NGO0744) and MutS (NGO1930) (32) were not found to be regulated by either glucose or L-lactate (Tables S1 and S2). In addition, host-derived L-lactate has been shown to induce micro-colony dispersal in the closely related species *Neisseria meningitidis* (33). Given these precedents and the up- and down-regulation of nuclease and adhesin genes, respectively, we examined whether L-lactate can impact biofilm formation in Ng. In this respect, analysis of crystal violet-stained biofilms showed that L-lactate greatly reduces biofilm formation compared to gonococci grown in a medium containing glucose (Fig. 6).

**TABLE 1:** Nuclease- and adhesin-encoding genes differentially regulated by L-lactate as determined by RNA-Seq

Locus tag (FA1090)	Functional annotation	Gene	Fold-change to L-lactate (10 mM/1 mM)	Significant (*P* < 0.05)	Fold-change to glucose (10 mM/1 mM)	Significant (*P* < 0.05)
NGO0042	Oligoribonuclease		3.0	Yes	1.4	No
NGO0259	Ribonuclease 3		2.9	Yes	1.9	No
NGO0300	Very short patch repair endonuclease		3.5	Yes	1.5	No
NGO0303	Restriction endonuclease		3.3	Yes	1.5	No
NGO0655	Exodeoxyribonuclease VII large subunit		3.0	Yes	1.2	No
NGO1561	Exodeoxyribonuclease III		2.8	Yes	1.2	No
NGO2181	Ribonuclease P protein component		2.7	Yes	1.6	No
NGO1392	Adhesin MafB	*mafB*	−2.5	Yes	−1.5	No
NGO1584	Adhesin MafA	*mafA*	−3.1	Yes	−1.8	Yes
NGO1585	Adhesin		−2.8	Yes	−1.7	Yes
NGO1971	Adhesin MafB	*mafB*	−1.5	Yes	−1.5	No

Gonococcal biofilms are characterized by a shift to anaerobic metabolism, having increased expression of *aniA, norB,* and *ccp* encoding nitrite reductase, nitric oxide reductase, and cytochrome c peroxidase, respectively, enzymes required for anaerobic respiration (34, 35). Due to the association of gonococcal biofilms with anaerobic respiration and the reduction of biofilm formation in the presence of L-lactate, we compared the L-lactate regulons with a previously determined anaerobic regulon (13) (Fig. 4b). This analysis revealed 60 co-regulated genes within the anaerobic condition and the exposure to L-lactate transcriptomes. Most of those genes are significantly correlated in an inverse manner in that the up-regulated genes by anaerobic conditions are down-regulated during growth in L-lactate and vice versa (Fig. 4e). An example of this type of association is as follows: while the anaerobic condition represses the expression of genes encoding respiratory enzymes such as subunits of NADH-ubiquinone reductase (NGO1414 to 1418), ribosomal proteins S3 (NGO1832), L22 (NGO1833), S19 (NGO1834), L4 (NGO1837) and L10 (NGO1841), and the RNA polymerase β subunits (NGO1850 and 1851), L-lactate enhances the expression of these genes. Different from L-lactate, there is no correlation in the fold-change regulation between the glucose and anaerobic regulons (Fig. 4h); thus, the above-mentioned co-regulation is specific to L-lactate. Overall, these results suggest that L-lactate acts as a signaling molecule promoting aerobic respiration, energy consumption, and biofilm dispersal, contrary to anaerobic conditions that promote a slower metabolism, and biofilm formation (13, 35).

### LctP can function as a unique pyruvate transporter

**Fig 7:**
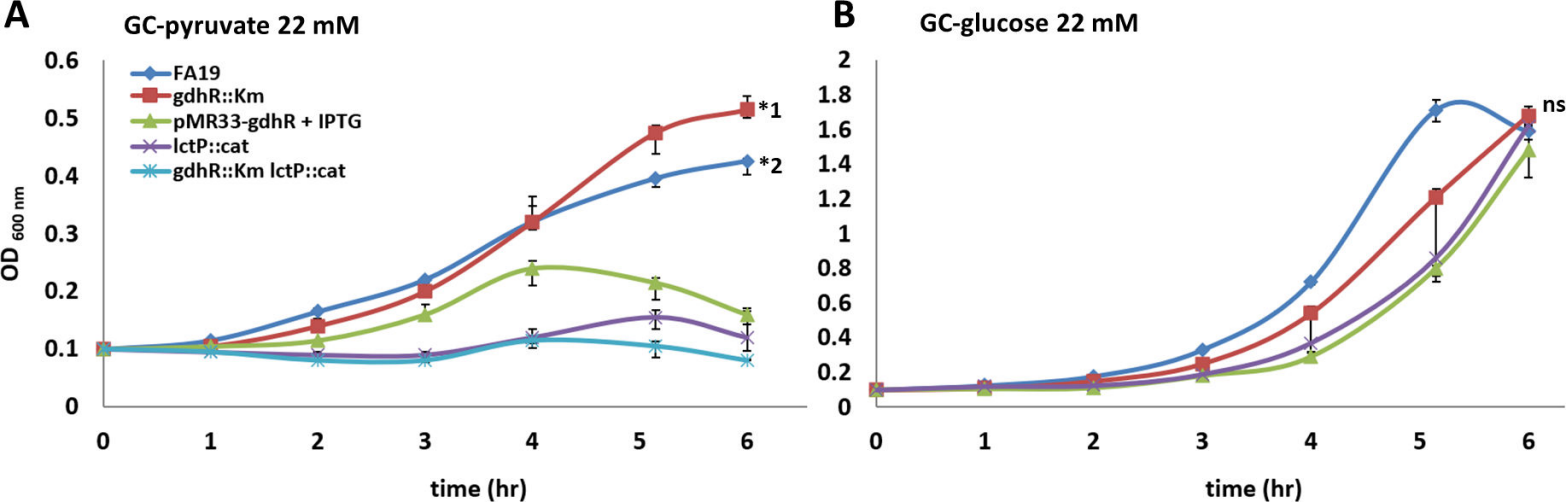
Gonococcal cells lacking *lctP* have a significant growth defect in GC-broth supplemented with pyruvate in replacement of glucose. Wild-type gonococcal strain FA19 and isogenic strains *gdhR* insertional mutant (FA19 *gdhR::kan*), complemented strain overexpressing *gdhR* in *trans* from the IPTG-inducible *lac* promoter (JC02), *lctP* insertional mutant (JC03), and double mutant *gdhR-lctP* (JC04) were grown in GC-broth supplemented with pyruvate in replacement of glucose in the Kellogg supplement (**A**) and with standard glucose-containing Kellogg supplement (**B**). Bacteria were grown with orbital shaking at 37°C for 6 h and the optical density (OD) at 600 nm was measured every hour. Data are represented as the median plus/minus the 75% and 25% percentiles (error bars), respectively, of three biological replicates. *1 statistically significant at 6 h from the WT using a nonparametric Mann-Whitney U-test (one-tailed *P* = 0.05). *2 statistically significant at 6 h from the rest of the strains using a U-test (one-tailed *P* = 0.05). ns non-significant statistical differences at 6 h from wild type using a one-tailed U-test.

Bacterial homologs of Ng LctP are not always specific transporters of L-lactate. For instance, the LldP transporter of *E. coli* can transport L-lactate, D-lactate, and glycolate (36). Ng can only efficiently rely upon glucose, L-lactate, and pyruvate as sole carbon sources (37). Pyruvate is a direct H_2_O_2_ scavenger using the following decarboxylation reaction: _3_HC-C^=O^-COO^−^ + H_2_O_2_ → CO_2_ + _3_HC-COO^−^ + H_2_O (38). Accordingly, we tested the possibility of LctP being involved in pyruvate transport. If true, this would add another mechanism by which it can protect gonococci against H_2_O_2_ killing. In support of this possibility, we found that loss of LctP rendered gonococci unable to grow in GC-broth containing pyruvate in replacement of glucose as the carbon source in the Kellogg’s supplement (Fig. 7A). By contrast, loss of GdhR, which significantly enhances LctP expression (18), increased the growth rate of gonococci compared to the wild-type strain at the late exponential phase, while ectopic overexpression of GdhR had the opposite effect (Fig. 7A). In addition, insertional inactivation of *lctP* in the *gdhR* mutant background completely abrogated Ng growth when pyruvate is the sole carbon source. In comparison, all of these isogenic strains showed the same growth rate in media containing glucose (Fig. 7B). These results suggest that LctP could be a unique pyruvate transporter in addition to L-lactate. However, the affinity of LctP for L-lactate might be higher than for pyruvate since L-lactate at the same concentration can increase the bacterial growth by 4.2-fold at late exponential phase compared to pyruvate (Fig. 7; Fig. S2).

## DISCUSSION

This work was stimulated by the recognition that Ng expression of LctP and its subsequent transport of L-lactate and downstream metabolism enhances bacterial resistance to hydrogen peroxide killing (18), in some strains resistance to killing by normal human serum (19, 20) and survival in human PMN leukocytes and cervical epithelial cells (39). The survival of Ng in PMNs, which produce high amounts of H_2_O_2_, can be explained in part by the fact that lactate derived from host cells enhances gonococcal metabolism (40, 41) and increases oxygen consumption by gonococci (26), all of which allow gonococci to effectively compete with PMN cells for the oxygen required to mount a respiratory burst and produce ROS. However, L-lactate induction of H_2_O_2_ resistance could not be explained when Ng grows alone in a bacteriological medium (18). To better understand this phenomenon, we have determined the gonococcal transcriptomic response to L-lactate and glucose in culture medium. L-lactate was found to regulate 37% of the gonococcal transcriptome while glucose only 9%. This result is consistent with the idea that L-lactate acts as a signaling molecule in pathogenic bacteria [reviewed in reference (42)]. Lactate, as the main product generated at the end of anaerobic respiration, has a ubiquitous presence in infection sites and has been found to enhance pathogenicity and host colonization of pathogenic bacteria such as *N. meningitidis* (43, 44), *Staphylococcus aureus* (45, 46), *Haemophilus influenzae* (47), and *Ng* (26, 48, 49). Thus, the extensive transcriptional regulatory activity exerted by L-lactate demonstrates the importance of this metabolite in regulating host–pathogen interactions during gonococcal infection.

**Fig 8:**
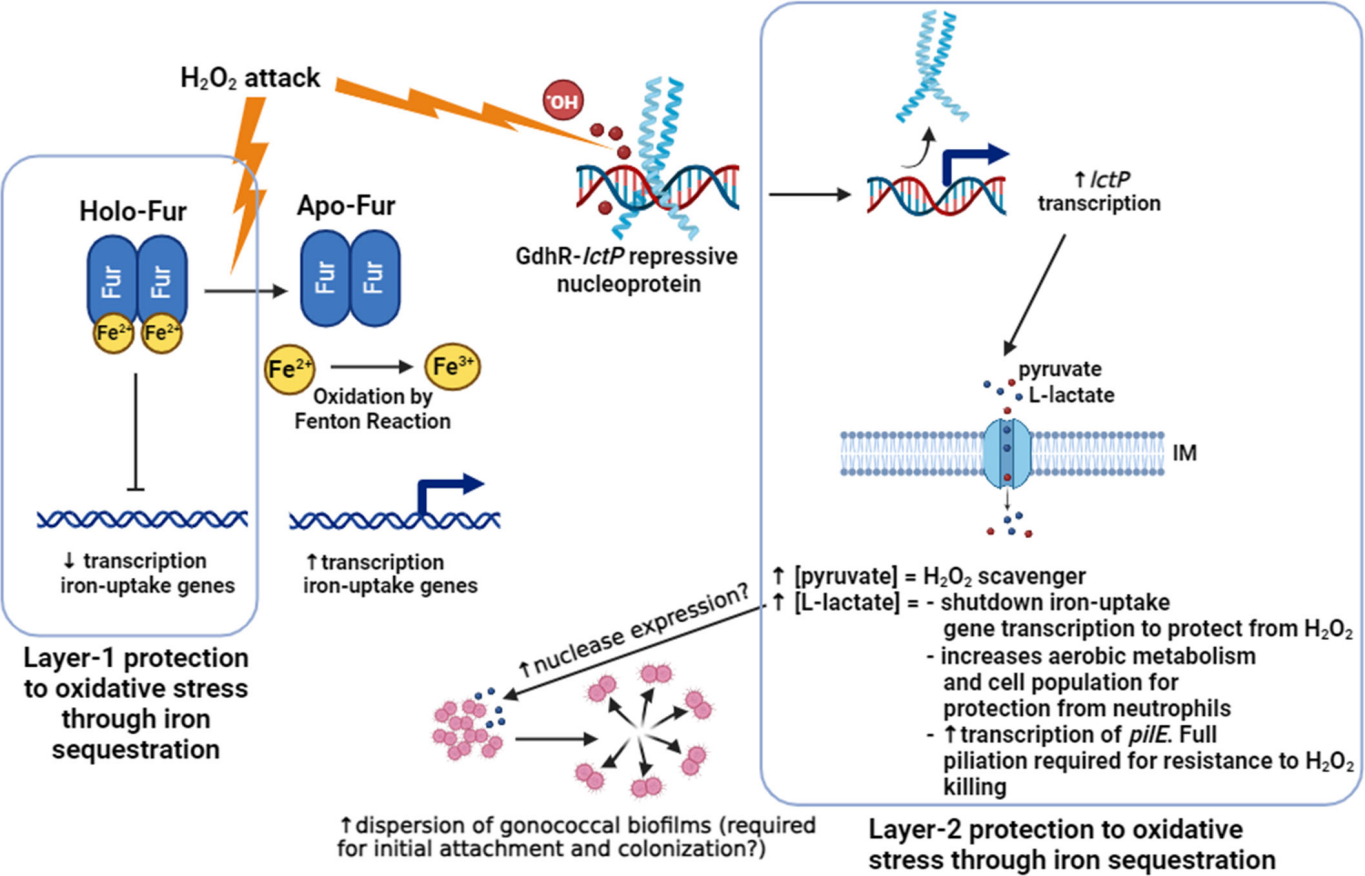
Proposed model for the mechanisms by which L-lactate transport and metabolism protect gonococcal cells from hydrogen peroxide-induced oxidative damage and promote survival within infection sites. Bacterial cells tightly control the level of free cytoplasmatic iron using the repressive action of the Holo-Fur dimer on iron uptake genes (11). This is because unbound ferrous iron catalyzes the conversion of the non-toxic H_2_O_2_ into damaging hydroxyl radicals by the Fenton reaction. We call the Fur protection Layer-1. However, in the presence of increasing concentration of H_2_O_2_, the ferrous iron clusters of Fur are oxidized to ferric iron by the Fenton reaction, resulting in the inability of Fur to repress the transcription of iron-uptake genes (14). We have shown that increasing concentrations of H_2_O_2_ in the presence of ferrous iron can attack and lead to a decrease in the formation of repressive GdhR-*lctP* nucleoprotein complexes, which increases *lctP* expression (18). Increased expression of LctP at the inner membrane (IM) can lead to an increased influx of L-lactate and pyruvate. Pyruvate is a direct H_2_O_2_ scavenger by undergoing a decarboxylation reaction (38) and protects bacterial cells from H_2_O_2_ killing (59). Increased L-lactate transport results in the repression of iron-uptake genes, which protects gonococcal cells from H_2_O_2_-induced oxidative damage. In addition, it increases the expression of genes encoding the tricarboxylic acid cycle, respiratory enzymes, and ribosomal proteins, which leads to an increase in oxygen consumption and acceleration of the metabolic activity allowing gonococci to effectively compete with neutrophils for oxygen and to resist the respiratory burst (26, 40, 41, 49). L-lactate induced transcription of *pilE,* encoding the main type IV pilus subunit, and piliation is required for resistance to H_2_O_2_- and neutrophil-mediated killing (60). We propose that the L-lactate regulon-induced effects constitute a layer-2 protection system from oxidative damage complimentary to the Fur regulation system and that it should take place at high levels of hydrogen peroxide exposure such as within the phagolysosome of polymorphonuclear cells. L-lactate was shown to effectively disrupt gonococcal biofilm formation, which could be important for the initial attachment and colonization of the genital sites (i.e., within the vaginal tract colonized by lactic acid-producing lactobacilli). The illustration was designed with BioRender.com.

In this work, we found that L-lactate exerts a suppressive effect on iron transport gene expression, thereby potentially limiting the cytoplasmic concentration of labile iron when L-lactate is available in the extracellular milieu. This phenomenon contrasts with the observed enhancement of iron transport gene expression in the presence of H_2_O_2_ (14), indicating a complex interplay between lactate and H_2_O_2_ in modulating iron homeostasis (Fig. 8). Importantly, Quillin et al. observed that gonococcal exposure to sub-lethal levels of H_2_O_2_ increases transcript abundance of TonB-dependent iron transporter genes *tbpA, tbpB*, *hpuB*, *fetA*, *fetB*, *tonB*, *exbB*, *exbD,* and *tdfG,* as well as the Fe-S cluster assembly and repair genes *iscS*, *iscA*, *iscU*, *erpA*, and their biosynthesis master regulator *iscR* (14). The iron-sulfur cluster-containing proteins constitute a diverse group of enzymes which are located ubiquitously throughout the bacterial cell and are vulnerable to oxidation (50). These genes encoding iron transporters and iron-sulfur cluster-containing proteins are under the regulation of intracellular iron levels and the Ferric Uptake Regulator (Fur), which regulates iron homeostasis (11). The enhanced transcription of this subset of genes in the face of a H_2_O_2_ attack seems counterintuitive given the potential for free cytoplasmic iron to catalyze the Fenton reaction. Varghese et al. have suggested that H_2_O_2_ may oxidize the ferrous ions within the iron-Fur complexes inactivating the repressor activity of Fur (27), which explains the H_2_O_2_-mediated increase in the transcription of iron transport genes. Thus, the L-lactate regulatory activity reported here could represent a secondary or containment mechanism for H_2_O_2_ protection, which can take over from the Fur regulation in controlling iron homeostasis and preventing the Fenton reaction from occurring, especially at high H_2_O_2_ concentrations (18). Moreover, L-lactate was shown to enhance the transcription of *pilE* (NGO_11105) encoding the main subunit of the gonococcal type IV pilus apparatus, which contributes to resistance against H_2_O_2_ killing by modulating intracellular labile iron (51). This suggests a link between lactate metabolism and the maintenance of surface structures that confer protection against oxidative damage. The redundant protection of Ng to oxidative damage through iron sequestration would be an important adaptive trait in the face of gonococcal survival within activated phagocytes or upon exposure to the commensal microbiota of the healthy vaginal tract, conditions in which gonococci encounter both L-lactate and high H_2_O_2_ production (26, 52, 53). In this respect, while most bacteria are very sensitive to hydrogen peroxide killing, for instance, *E. coli* and *V. cholerae* cultures are killed *in vitro* at levels of 1–3 mM of H_2_O_2_ (54–56), we have detected *in vitro* survival of wild-type Ng in H_2_O_2_ up to 50 mM (18). In addition, the simultaneous presence of the OxyR and PerR transcriptional regulatory systems, which control the expression of multiple ROS detoxifying enzymes, in a bacterium is rare, happening only in Ng and in two *Streptomyces* spp (57). A limitation of our analysis is that the L-lactate regulon was studied in strain FA19 and *in vitro*, while other strains have been used for determining the H_2_O_2_, iron, and anaerobic regulons. However, we have shown that the *lctP*-mediated protection against H_2_O_2_ killing and the *lctP* regulation are conserved traits in different Ng laboratory strains (18, 58). Furthermore, transcriptomic models of Ng exposed to PMNs have suggested that Ng imports pyruvate and D-lactate in the presence of PMNs, which support its growth independently of the glycolytic pathway (15). Lastly, alignment of the nucleotide sequences for critical regulators of these systems (GdhR for *lctP* regulation, Fur for iron acquisition regulation, and OxyR for regulation susceptibility to H_2_O_2_ killing) possessed by the strains (FA19, FA1090, and F62) used in these different regulon studies showed no differences (Fig. S3).

The involvement of LctP as a possible pyruvate transporter adds another dimension to this relationship, potentially explaining the observed association between LctP expression and H_2_O_2_ resistance. Pyruvate, the oxidized α-keto acid form of lactate, is a direct H_2_O_2_ scavenger (61) and one of the few sugars used by Ng (37). The transport of major sugars such as lactate and pyruvate by LctP underscores the importance of LctP as a virulent factor for Ng. We assessed the degree of sequence conservation among the *lctP* loci of 9960 strains of Ng in the public database PubMLST (62) (49.5% of the current repository as of 03/04/2024). We found that there were only three missense mutations with significant frequency percent (I244V 4%, G289S 10%, and G463D 4%), while the rest had less than 0.1% frequency, and any nonsense mutations were found (Table S4). We found only one frameshift mutation, but this is in a poly adenosine tract (8As) and could be a sequencing artifact common in such polynucleotide tracts. In addition, there were only 28 mutations (0.3%) in the intergenic region with none of them impacting the sigma-70 promoter or the transcriptional start site of *lctP*. Thus, the conservation of *lctP* alleles among clinical strains further emphasizes its significance in the adaptive response of Ng to host environmental challenges.

As we had reported before (18), we found that *lctP* expression is subjected to glucose repression. Although we have not found a regulatory mechanism to explain this carbon catabolite repression event, we could confirm that it is not mediated through the PTS gene *ptsK* (NGO0314) (18). In Ng, there is not a complete PTS systems connected to glucose transport that sense the energetic state of the cell (63). Recently in the genetically close relative *N. meningitidis,* the transport of glucose was shown to be carried out by the glucose permease *glcP*, encoding a major facilitator superfamily (MFS) transporter (64). To confirm whether glucose transport in Ng occurs only *via glcP* as in *N. meningitidis,* we performed an insertional-deletion mutation in the Ng *glcP* homolog (NGO0142) using the neomycin phosphotransferase gene *nptII* (Fig. S4). We found that *glcP* mutants of Ng cannot grow in GC broth supplemented with glucose; however, the replacement of glucose by L-lactate in Kellogg supplement I fully restores the ability of the mutants to grow to wild-type levels (Fig. S4). These results highlight the importance of LctP and GlcP for sugar uptake and metabolism during gonococcal infection, which make them attractive targets for the development of anti-virulence strategies.

We found an inverse association between the co-regulated genes among the L-lactate transcriptome and the anaerobic regulon. When Ng is exposed to H_2_O_2_, the gene *aniA* required for anaerobic respiration and the genes encoding the nitrate-responsive two-component regulatory system NarQ-NarP responsible for the transcriptional activation of *aniA* have decreased transcript abundance (14). In fact, several other genes and regulators involved in oxygen sensing, cellular respiration, denitrification, and anaerobiosis were found to display a co-regulation between the H_2_O_2_ and the anaerobic regulons, which suggests that down-regulation of anaerobic respiration serves to protect *N. gonorrhoeae* from oxidative damage (14). Similarly, our data showed the L-lactate-mediated induction of aerobic respiratory enzymes, which are essential for H_2_O_2_ resistance *in vivo*. In gonococcal biofilms, there is a shift toward anaerobic respiration (34, 35), and, accordingly, we found an antagonistic effect of L-lactate on gonococcal biofilm formation. Since L-lactate transport and metabolism yield a transcriptionally opposite effect to the anaerobic respiration and to biofilm formation, we suggest that Ng evolved to sense L-lactate to determine its surroundings and to use this carbohydrate to counteract the toxic effects of H_2_O_2_. For instance, the healthy vaginal tract is dominated by commensal *Lactobacillus* spp. that produces high amounts of bactericidal lactic acid and H_2_O_2_ (65, 66). The L-lactate-mediated dispersal of biofilms and induction of *pilE* transcription could also promote the initial attachment required for colonization of the vaginal tract. This could be particularly important since Ng could arrive at the vaginal tract in the form of biofilms since gonococci exposed to seminal fluids increase bacterial aggregation and can form three-dimensional biofilms (67). As Ng moves upper into the female genital tract such as the endocervix, which is further from the external (aerobic) environment, then biofilm formation could be the more predominant lifestyle. Regarding this, Ng has been shown to form biofilms during cervical infection (68) and *in vitro* on primary human cervical cells (12). Such repeated transitions between the biofilm and planktonic single-cell living lifestyles are required for effective colonization, dissemination, and transmission in the lifecycle of the cholera-causing bacterium *Vibrio cholerae* (69).

The gonococcal genetic island (GGI) contains several genes encoding a Type 4 secretion system (T4SS) that translocates single-stranded DNA into the extracellular space (70). This secreted DNA nucleates and helps to build up the early phase of gonococcal biofilm formation (71). We found that lactate decreased the expression of several genes encoding the T4SS such as *atlA,* which is similar to endolysins from bacteriophages (72), and *traC*, *traF,* and *traN* encoding components of the T4SS core complex (73) (Table S5). Thus, a reduction in secreted DNA due to decreased expression of T4SS genes could impact biofilm formation by gonococci in a lactate-rich environment. In addition, it is suggested that the T4SS could facilitate iron transport in a Ton complex-independent manner (74), which could constitute another pathway of iron sequestration exerted by the L-lactate transcriptional regulatory activity evidenced in this work.

An additional consideration for the ability of Ng to survive during cervical infection is the ability of invading bacteria to resist the bactericidal action of normal human serum (NHS) either constitutively or by an inducible mechanism that can involve sialylation of the lipooligosaccharide (LOS) [reviewed in reference (75)]. In this regard, our work employed strain FA19 which expresses a constitutive serum resistance phenotype due to possession of the major outer membrane protein PorB1A (76). We note, however, that the lactate and glucose regulons reported herein did not contain genes involved in LOS biosynthesis as defined by the set criteria of ≥2-fold-change in expression (Tables S1 and S2). Further work on lactate utilization and regulated genes in strains displaying an inducible NHS-resistant phenotype is needed to further understand the influence of lactate on the ability of Ng to escape killing by NHS possibly by changes in the structure or levels of LOS.

In conclusion, this work reveals the intricate interplay between L-lactate metabolism, iron homeostasis, stress response, and biofilm formation in Ng, contributing to our understanding of gonococcal pathogenesis and highlighting potential targets for future therapeutic interventions. We stress that our findings and those of others (19, 39) support the proposition first put forth by the late Harry Smith (22, 77) that the ability of Ng to transport and utilize lactate is a critical aspect of its virulence potential.

## MATERIALS AND METHODS

### Strains and media

*N. gonorrhoeae* laboratory-maintained strains FA19 with an *rpsL* mutation at codon 43 resulting in a single amino acid change (K to R) conferring resistance to streptomycin (Str^R^) (78) and F62 (79) were used in this study. FA19 Str^R^ isogenic mutants strains FA19 *gdhR::kan*, JC2 (*gdhR::kan* complement with pMR33-*gdhR*), JC3 (*lctP::cat*), and JC4 (*gdhR::kan-lctP::cat*) were previously constructed (18). Gonococcal strains were grown overnight at 37°C under 5% (vol/vol) CO_2_ on GC agar plates containing Kellogg’s supplements I and II (58). When indicated, glucose in the supplement I was replaced by L-lactate or pyruvate at indicated concentrations. Growth in liquid medium was at 37°C with agitation (225 r.p.m.) in GC broth containing Kellogg’s supplements I and II and 0.042% (wt/v) sodium bicarbonate.

### Extraction of total RNA and qRT-PCR

*N. gonorrhoeae* cultures were grown in GC broth at 37°C with agitation to the mid-exponential phase. At 4 h, 2.5 mL samples were centrifuged, resuspended in 200 µL RNA*later* solution (Ambion), and incubated for 10 min on ice. Total RNA extraction was conducted using the RNeasy mini kit (Qiagen) following the manufacturer’s protocol. Contamination with gDNA was removed using the Turbo DNA-free Kit (Invitrogen). DNase I-digested total RNA samples were further cleaned up and concentrated using the RNeasy MinElute Cleanup Kit and sent for sequencing at the UAB Heflin Center for Genomic Science. The integrity of the purified RNA samples was determined by formaldehyde agarose gel electrophoresis as described before (63).

For qRT-PCR, the DNase I-digested total RNA samples were reverse transcribed using the QuantiTect Reverse Transcription Kit (QIAGEN). Real-Time PCR was conducted using the IQ SYBR Green Supermix and a CFX Connect Real-Time System (Bio-Rad Laboratories). Relative expression values were calculated as $2^{\left(CT_{references}-CT_{target}\right)}$, where CT is the fractional threshold cycle. Fold-change regulation was calculated by dividing the larger relative expression value over the smaller value between the 1 and 10 mM sugar variations. The level of 16S rRNA was used as an internal reference. Oligonucleotide primer pairs used to quantify relative mRNA levels of the subset of analyzed genes are in Table S6.

### RNA-Seq and bioinformatics analysis

RNA-Sequencing was performed on the Illumina NextSeq 500 following the manufacturer recommendations (Illumina Inc., San Diego, CA) as described before (18). For the RNA-Seq bioinformatics analysis, software deposited in the public ABIMS Galaxy tool shed (Station Biologique de Roscoff-CNRS-Sorbonne University) was used. Briefly, single-end fastq files generated by the sequencing platform were aligned to the *N. gonorrhoeae* FA19 genome (GenBank assembly accession: GCA_000273665.1) using the Tophat2 algorithm to generate BAM files. Transcript differential expression between the conditions (*n* = 2) was determined using the aligned BAM files and the Cuffdiff algorithm. Finally, the BAM files, the Tophat2 alignment rates, and the RNA-Seq laboratory and bioinformatics methods were deposited in the Gene Expression Omnibus (GEO) (64) with GEO series accession number GSE148774.

### Static gonococcal biofilm formation

*N. gonorrhoeae* strains were grown overnight in GC-agar plates and the biomass was collected to make bacterial suspensions at OD600nm 0.1 in GC-broth supplemented with either glucose or L-lactate at 22 mM. One hundred microliters was incubated statically in 96-well polystyrene plates at 37°C, 5% (vol/vol) CO_2_ for 24 h. After incubation, the OD_600nm_ of the plates was collected, planktonic cells were discarded, and the wells were washed once with 100 µL filter sterilized PBS1X. One hundred microliter of 0.1% (wt/vol) crystal violet was added to the wells and incubated for 30 min in the dark, and the wells were subsequently washed four times with 200 µL of PBS1X. The crystal violet attached to the biofilms was resolubilized with 120 µL of DMSO and the OD_570nm_ was collected in a 96-well plate reader. Wells containing GC-broth only were used as blank measurements. Finally, the biofilm formation of each well was calculated as the ratio OD_570_/OD_600_.

### Statistical analyses

All statistical analyses were done using the GraphPad Prism 5.0 software (GraphPad, San Diego, CA). For small sample size data when a normality test cannot be done the data were analyzed using the nonparametric test Mann-Whitney to compare the medians. The nonparametric Spearman test was used to analyze the correlation between data sets.

## Data Availability

The data sets that supported the findings of this study are available from the corresponding author upon request.

## SUPPLEMENTAL MATERIAL

Figure S1 mbio.01761-24-s0001.docx Gonococcal growth curves under the conditions tested for RNA-Seq analysis.

Figure S2 mbio.01761-24-s0002.docx Growth of gonococcal strains in GC-L-lactate broth.

Figure S3 mbio.01761-24-s0003.docx Nucleotide alignments of transcriptional regulators.

Figure S4 mbio.01761-24-s0004.docx Gonococcal *glcP* mutant construction and phenotypic confirmation.

Table S1 mbio.01761-24-s0005.xlsx Lactate regulated genes.

Table S2 mbio.01761-24-s0006.xlsx Glucose regulated genes.

Table S3 mbio.01761-24-s0007.docx Regulation of iron and other metal transport genes by glucose and L-lactate.

Table S4 mbio.01761-24-s0008.xlsx *lctP* alignments.

Table S5 mbio.01761-24-s0009.docx Regulation of genes in the gonococcal genetic island by L-lactate and glucose.

Table S6 mbio.01761-24-s0010.docx Oligonucleotide primers used in this study.

ASM does not own the copyrights to Supplemental Material that may be linked to, or accessed through, an article. The authors have granted ASM a non-exclusive, world-wide license to publish the Supplemental Material files. Please contact the corresponding author directly for reuse.
