# Supplementary material for: Transcriptional responses of Neisseria gonorrhoeae to glucose and lactate: implications for resistance to oxidative damage and biofilm formation
Source: mBio. 2024 Jul 16;15(8):e01761-24. doi: 10.1128/mbio.01761-24 (PMC11323468; doi:10.1128/mbio.01761-24)

**Fig S1. Gonococcal growth curves under the conditions tested for RNA-Seq analysis.** Gonococcal strain FA19 was grown in GC-broth supplemented with either 1 or 10 mM of L-lactate or glucose and with a fix concentration of 1.5 mM glucose to assess the L-lactate variation and a fix 3 mM L-lactate to assess the glucose variation. 2.5 mL of cultures were collected at 4 hr for RNA extraction at the mid-exponential phase (circle points). There were not statistical differences in OD600nm between glucose curve collection points (*p* = 0.07) or in the L-lactate curve (*p* = 0.19) using non-paired two-tail T-tests.


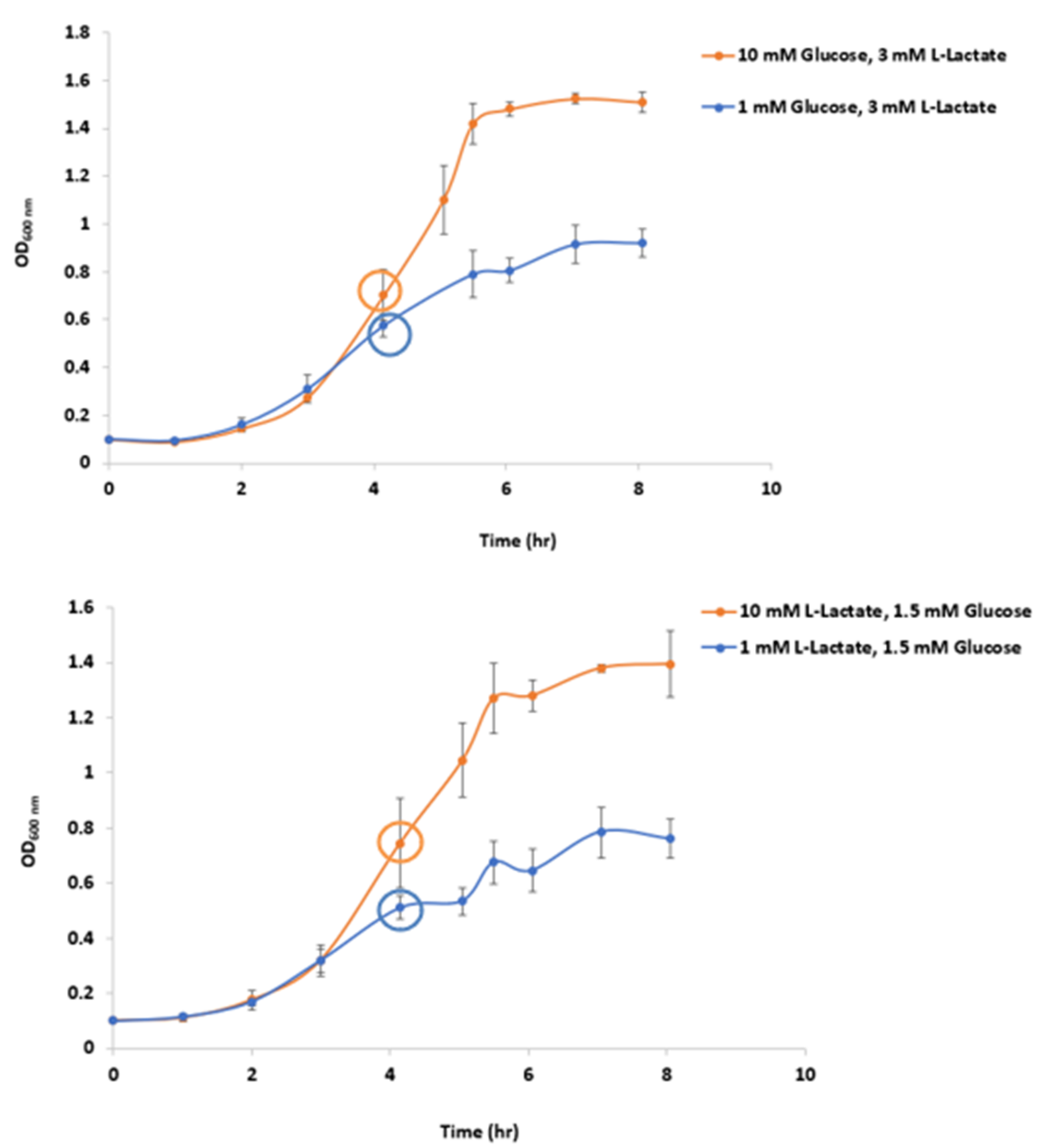

Supplement: Figure S1 — Gonococcal growth curves under the conditions tested for RNA-Seq analysis. [file mbio.01761-24-s0001.docx]
