# Supplementary material for: Transcriptional responses of Neisseria gonorrhoeae to glucose and lactate: implications for resistance to oxidative damage and biofilm formation
Source: mBio. 2024 Jul 16;15(8):e01761-24. doi: 10.1128/mbio.01761-24 (PMC11323468; doi:10.1128/mbio.01761-24)

**Fig S2. Growth curve of gonococcal strains in GC-L-lactate broth.** Wild-type gonococcal strain FA19 and the isogenic strains *gdhR* insertional mutant (FA19 *gdhR*::*kan*), complemented strain overexpressing *gdhR* in trans from the IPTG-inducible *lac* promoter (JC02), *lctP* insertional mutant (JC03) and double mutant *gdhR*-*lctP* (JC04) were grown in GC-broth supplemented with L-lactate in replacement of glucose in the Kellogg supplement. Bacteria were grown with orbital shaking at 37^o^C for 5 hr and the optical density (OD) at 600nm was measure every hour.


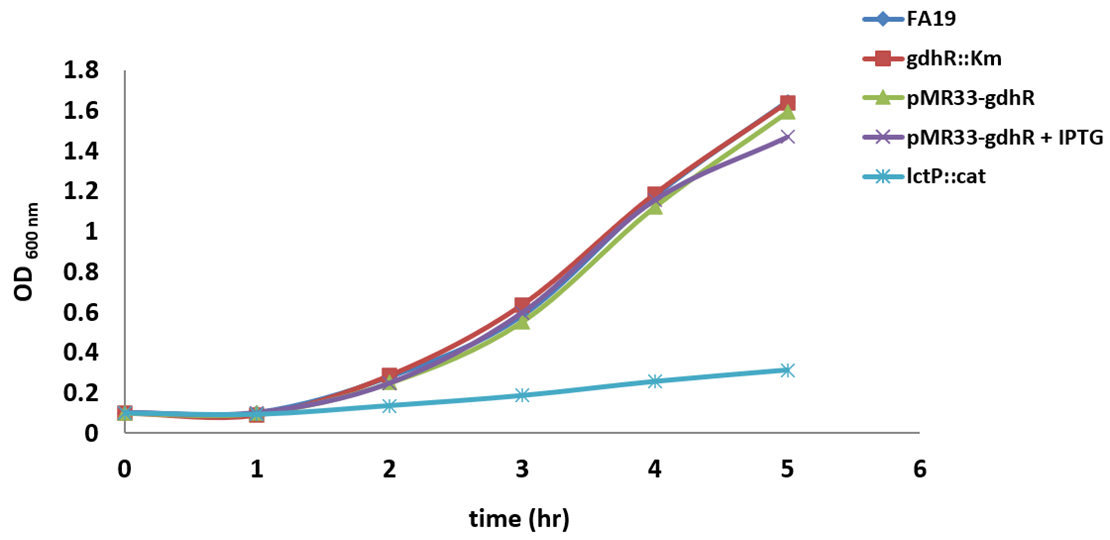

Supplement: Figure S2 — Growth of gonococcal strains in GC-L-lactate broth. [file mbio.01761-24-s0002.docx]
