# Supplementary material for: Transcriptional responses of Neisseria gonorrhoeae to glucose and lactate: implications for resistance to oxidative damage and biofilm formation
Source: mBio. 2024 Jul 16;15(8):e01761-24. doi: 10.1128/mbio.01761-24 (PMC11323468; doi:10.1128/mbio.01761-24)
Supplement: Figure S3 — Nucleotide alignments of transcriptional regulators. [file mbio.01761-24-s0003.docx]

**Fig. S3. Alignment of the nucleotide sequence of *fur, oxyR* and *gdhR* among laboratory strains FA19, FA1090 and F62.** The nucleotide alignments of transcriptional regulators from three gonococcal strains are shown. The nucleotide sequence of *gdhR* among these strains are 100% identical as shown previously (Ayala JC, Schmerer MW, Kersh EN, Unemo M, **Shafer WM**. 2022. Gonococcal clinical strains bearing a common *gdhR* single nucleotide polymorphism that results in enhanced expression of the virulence gene *lctP* frequently possess a *mtrR* promoter mutation that decreases antibiotic susceptibility*. mBio*. 8:e0027622. doi: 10.1128/mbio.00276-22.)

**oxyR alignment**

Consensus ATGACCTTAACCGAATTGCGGTACATCGTCGCAGTCGCCCAAGAACGTCATTTCGGCCGG 60

OxyR_FA1090 ............................................................ 60

OxyR-FA19 ............................................................ 60

OxyR_F62 ............................................................ 60

Consensus GCGGCGCGGCGTTGTTTTGTCAGCCAGCCCACTTTGTCTATTGCCATTAAGAAATTGGAA 120

OxyR_FA1090 ............................................................ 120

OxyR-FA19 ............................................................ 120

OxyR_F62 ............................................................ 120

Consensus GAAGAGCTTGCCGTCTCTTTGTTTGACCGGAGCAGCAACGATATTATTACGACCGAGGCG 180

OxyR_FA1090 ............................................................ 180

OxyR-FA19 ............................................................ 180

OxyR_F62 ............................................................ 180

Consensus GGGGAACGTATCGTTGCACAGGCGCGTAAGGTATTGAAAGAGGCGGAGCTTATCAGGCAT 240

OxyR_FA1090 ............................................................ 240

OxyR-FA19 ............................................................ 240

OxyR_F62 ............................................................ 240

Consensus TTGGCAAATGAAGAACAAAACGAGCTGGAGGGTGCGTTCAAACTCGGGCTGATTTTTACG 300

OxyR_FA1090 ............................................................ 300

OxyR-FA19 ............................................................ 300

OxyR_F62 ............................................................ 300

Consensus GTTGCGCCATACCTGCTGCCGAAACTGATTGTCTCGTTGCGCCGTACTGCACCGAAAATG 360

OxyR_FA1090 ............................................................ 360

OxyR-FA19 ............................................................ 360

OxyR_F62 ............................................................ 360

Consensus CCTTTGATGTTGGAAGAGAATTACACGCATACTTTGACCGAGTCGCTCAAACGCGGGGAC 420

OxyR_FA1090 ............................................................ 420

OxyR-FA19 ............................................................ 420

OxyR_F62 ............................................................ 420

Consensus GTTGACGCGATTATCGTTGCCGAACCGTTTCAAGAGCCGGGCATTGTTACCGAACCCTTG 480

OxyR_FA1090 ............................................................ 480

OxyR-FA19 ............................................................ 480

OxyR_F62 ............................................................ 480

Consensus TATGACGAACCGTTTTTCGTGATTGTCCCGAAAGGGCATTCATTTGAGGAACTGGATGCC 540

OxyR_FA1090 ............................................................ 540

OxyR-FA19 ............................................................ 540

OxyR_F62 ............................................................ 540

Consensus GTTTCGCCCCGGATGCTGGGTGAGGAGCAGGTTTTGCTGCTGACGGAAGGCAACTGTATG 600

OxyR_FA1090 ............................................................ 600

OxyR-FA19 ............................................................ 600

OxyR_F62 ............................................................ 600

Consensus CGGGATCAGGTACTCTCAAGCTGTTCCGAATTGGCGGCGAAACAGCGCATACAGGGGCTG 660

OxyR_FA1090 ............................................................ 660

OxyR-FA19 ............................................................ 660

OxyR_F62 ............................................................ 660

Consensus ACCAATACATTGCAGGGCAGCTCGATCAATACAATCCGCCATATGGTCGCCAGCGGTTTG 720

OxyR_FA1090 ............................................................ 720

OxyR-FA19 ............................................................ 720

OxyR_F62 ............................................................ 720

Consensus GCAATCAGCGTGTTGCCGGCAACCGCGCTGACCGAGAACGATCATATGCTGTTCAGCATT 780

OxyR_FA1090 ............................................................ 780

OxyR-FA19 ............................................................ 780

OxyR_F62 ............................................................ 780

Consensus ATTCCGTTTGAAGGTACGCCGCCAAGCCGGCGGGTCGTATTGGCGTATCGCCGTAATTTT 840

OxyR_FA1090 ............................................................ 840

OxyR-FA19 ............................................................ 840

OxyR_F62 ............................................................ 840

Consensus GTCCGTCCGAAGGCGTTGTCGGCGATGAAGGCGGCGATTATGCAGTCGCAGCTTCACGGG 900

OxyR_FA1090 ............................................................ 900

OxyR-FA19 ............................................................ 900

OxyR_F62 ............................................................ 900

Consensus GTAAGTTTTATCCACGACTAG 921

OxyR_FA1090 ..................... 921

OxyR-FA19 ..................... 921

OxyR_F62 ..................... 921

**Fur alignment**

Consensus ATGGAAAAATTCAGCAACATTGCGCAACTGAAAGACAGCGGTCTGAAGGTTACCGGCCCG 60

fur-FA1090 ............................................................ 60

fur_FA19 ............................................................ 60

fur_F62 ............................................................ 60

Consensus CGTTTGAAGATTTTGGATTTGTTCGAGAAGCACGCGGAAGAGCATTTGAGTGCGGAAGAT 120

fur-FA1090 ............................................................ 120

fur_FA19 ............................................................ 120

fur_F62 ............................................................ 120

Consensus GTGTACCGCATTCTGCTGGAAGAGGGCGTGGAAATCGGTGTGGCGACGATTTACCGCGTG 180

fur-FA1090 ............................................................ 180

fur_FA19 ............................................................ 180

fur_F62 ............................................................ 180

Consensus CTGACGCAGTTCGAGCAGGCGGGCATTCTGCAACGCCACCATTTTGAAACGGGCAAGGCG 240

fur-FA1090 ............................................................ 240

fur_FA19 ............................................................ 240

fur_F62 ............................................................ 240

Consensus GTTTATGAGTTGGACAAGGGCGACCACCACGACCATATCGTCTGCGTGAAGTGCGGCGAG 300

fur-FA1090 ............................................................ 300

fur_FA19 ............................................................ 300

fur_F62 ............................................................ 300

Consensus GTAACGGAATTCCACAATCCCGAAATCGAAGCCCTGCAAGACAAAATCGCCGAGGAAAAC 360

fur-FA1090 ............................................................ 360

fur_FA19 ............................................................ 360

fur_F62 ............................................................ 360

Consensus GGCTACCGCATCGTCGATCACGCGCTTTATATGTACGGCGTGTGCAGCGACTGTCAGGCC 420

fur-FA1090 ............................................................ 420

fur_FA19 ............................................................ 420

fur_F62 ............................................................ 420

Consensus AAGGGCAAACGTTAA 435

fur-FA1090 ............... 435

fur_FA19 ............... 435

fur_F62 ............... 435
