# Supplementary material for: Transcriptional responses of Neisseria gonorrhoeae to glucose and lactate: implications for resistance to oxidative damage and biofilm formation
Source: mBio. 2024 Jul 16;15(8):e01761-24. doi: 10.1128/mbio.01761-24 (PMC11323468; doi:10.1128/mbio.01761-24)

**Fig S4. Gonococcal *glcP* mutant construction and phenotypic confirmation.** To construct a gonococcal *glcP* mutant an insertional deletion mutation was performed using a plasmid construct (pglcP::kan) obtained from H.S. Seifert ([Ongoing FA1090 Mutant List – GC Knockout Library Database (northwestern.edu)](https://sites.northwestern.edu/gcmutantdatabase/resources/)). This plasmid construct contains a deletion of the *glcP* (NGO0142) coding sequence from nucleotides 16 to 1209, and a 1034 bp insertion cassette encoding the neomycin phosphotransferase gene *nptII* in replacement of the deleted region. Transformation of strain FA1090 (1-81-S2) P+ non-variant opaless + *opaD* non-variant [[80](../../wshafer/Desktop/2024%20MBio%20Ayala%20et%20al/Ayala%20Final%20files%20for%202024%20mBio/Ayala%20et.%20al.%20mBio%202024%20MPJ%20Comments%20tracked-WMS-2.docx#_ENREF_80)] was carried out with 28 ng of pglcP::kan using the spot agar transformation method described before [[81](../../wshafer/Desktop/2024%20MBio%20Ayala%20et%20al/Ayala%20Final%20files%20for%202024%20mBio/Ayala%20et.%20al.%20mBio%202024%20MPJ%20Comments%20tracked-WMS-2.docx#_ENREF_81)]. Transformants (strain JC105) were selected in GC-agar plates containing 50 µg/mL of kanamycin. **(A)** To confirm the deletion-insertion mutation a PCR was carried out with primers glcP-Fw (GCAATATTCCGACACCACGAAAGG) and glcP-Rv (TTCTCAAGAAAAAGATTATTTGTCCGC) and the product was visualized on an agarose DNA gel. Lane-1 1Kb Plus DNA ladder. Lane-2 Wild type FA1090 strain. Lane-3 mutant strain JC105 clone-7 (FA1090 P+ nv opaless +*opaD* nv *glcP*::kan). **(B-C)** To confirm phenotypes, selected *glcP* mutants were grown in GC broth (37 ^o^C, 225 rpm) supplemented with either glucose or L-lactate as sole carbon sources.

**REFERENCES**

80. Ball, L.M. and A.K. Criss, *Constitutively Opa-expressing and Opa-deficient neisseria gonorrhoeae strains differentially stimulate and survive exposure to human neutrophils.* J Bacteriol, 2013. **195**(13): p. 2982-90.

81. Dillard, J.P., *Genetic Manipulation of Neisseria gonorrhoeae.* Curr Protoc Microbiol, 2011. **Chapter 4**: p. Unit4A 2.


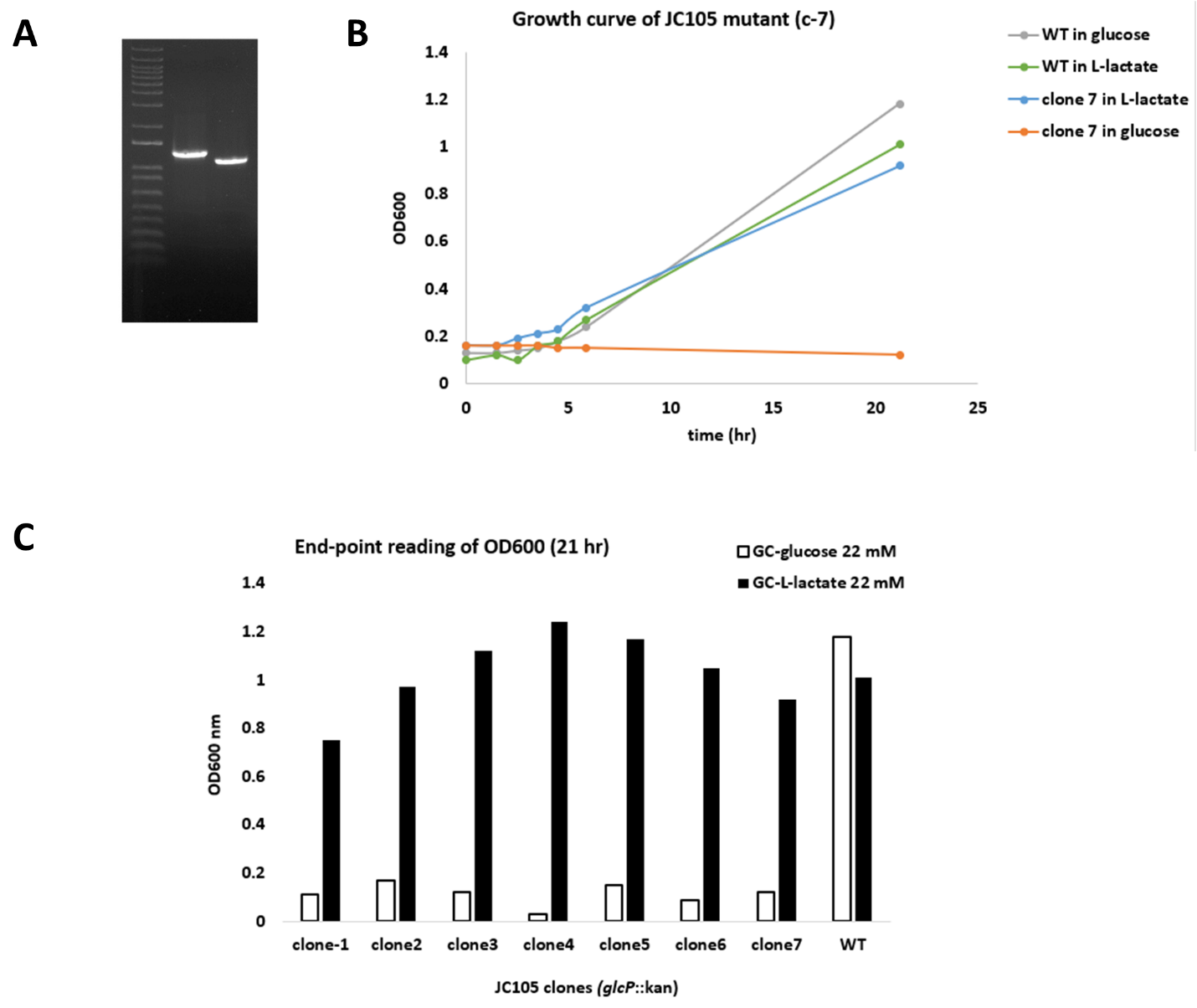

Supplement: Figure S4 — Gonococcal glcP mutant construction and phenotypic confirmation. [file mbio.01761-24-s0004.docx]
