# Supplementary material for: Transcriptional responses of Neisseria gonorrhoeae to glucose and lactate: implications for resistance to oxidative damage and biofilm formation
Source: mBio. 2024 Jul 16;15(8):e01761-24. doi: 10.1128/mbio.01761-24 (PMC11323468; doi:10.1128/mbio.01761-24)
Supplement: Table S3 — Regulation of iron and other metal transport genes by glucose and L-lactate. [file mbio.01761-24-s0007.docx]

**Table S3. Regulation of iron and other metal transport genes by glucose and L-lactate**

| **Locus tag (FA1090)** | **Functional annotation** | **Gene** | **Substrate** | **Fold-change to L-lactate**  **(10 mM/1 mM)** | **Significant (*p* < 0.05)** | **Fold-change to glucose**  **(10 mM/1 mM)** | **Significant (*p* < 0.05)** |
| --- | --- | --- | --- | --- | --- | --- | --- |
| NGO1377 | biopolymer transporter ExbD | *exbD* |  | -2.5 | Yes | -1.4 | No |
| NGO1379 | protein TonB | *tonB* |  | -2.5 | Yes | -1.2 | No |
| NGO1378 | biopolymer transporter ExbB | *exbB* |  | -2.6 | Yes | -1.2 | No |
| NGO1495 | transferrin-binding protein 1 | *tbpA* | Transferrin | -3.1 | Yes | -1.7 | Yes |
| NGO1496 | transferrin-binding protein 2 | *tbpB* | Transferrin | -3.2 | Yes | -1.8 | Yes |
| NGO0217 | iron ABC transporter substrate binding protein | *fbpC* | iron | -2.7 | Yes | -1.8 | No |
| NGO2092 | iron ABC transporter substrate binding protein | *fetB* | Enterobactin (siderophore) | -2.2 | Yes | -2.0 | Yes |
| NGO2093 | TonB-dependent siderophore receptor | *fetA* | Enterobactin (siderophore) | -2.5 | Yes | -1.7 | No |
| NGEG_RS0102070 (in FA19) | Lactoferrin binding protein A | *lbpA* | Lactoferrin | -1.8 | Yes | -1.2 | No |
| NGO0168 | Zinc/Mn ABC transporter | *znuA/mntC* | Zn/Mn transition metals | -2.8 | Yes | -2.3 | Yes |
