# Supplementary material for: Transcriptional responses of Neisseria gonorrhoeae to glucose and lactate: implications for resistance to oxidative damage and biofilm formation
Source: mBio. 2024 Jul 16;15(8):e01761-24. doi: 10.1128/mbio.01761-24 (PMC11323468; doi:10.1128/mbio.01761-24)
Supplement: Table S5 — Regulation of genes in the gonococcal genetic island by L-lactate and glucose. [file mbio.01761-24-s0009.docx]

**Table S5.**

**Regulation of genes within the Gonococcal Genetic Island by lactate and glucose^a^**

| **FA19 locus tag** | **gene name** | **Fold-change to lactate** | **significant** | **Fold-change to glucose** | **significant** |
| --- | --- | --- | --- | --- | --- |
| NGEG_RS0104865 | *yaa* | -1.344703668 | no | -1.024757633 | no |
| NGEG_RS0104870 | *traD* | -1.130498997 | no | -1.025535238 | no |
| NGEG_RS0104875 | *traI* | -1.441805268 | no | -1.351217418 | no |
| NGEG_RS0104880 | *yaf* | -1.209969272 | no | -1.023492178 | no |
| NGEG_RS14460 | *ltgX* | -1.710013605 | yes | -1.405710662 | no |
| NGEG_RS0104890 | *yag* | -1.560666093 | no | -1.205111465 | no |
| NGEG_RS0104895 | *traA* | -1.025413712 | no | 1.181912474 | no |
| NGEG_RS0104900 | *traL* | 1.235478105 | no | -1.564865874 | no |
| NGEG_RS12705 | *traE* | -1.451982517 | no | -1.390501091 | no |
| NGEG_RS0104910 | *traK* | -1.642152615 | no | -1.679748978 | no |
| NGEG_RS0104915 | *traB* | -1.683646436 | yes | -1.387536385 | no |
| NGEG_RS0104920 | *dsbC* | -1.165028494 | no | -1.35622887 | no |
| NGEG_RS0104925 | *traV* | -1.517243143 | no | -1.508479364 | no |
| NGEG_RS0104930 | *traC* | -2.020237186 | yes | -1.535063961 | no |
| NGEG_RS0104935 | *ybe* | -1.510320417 | no | -1.558754079 | no |
| NGEG_RS0104940 | *trbI* | -1.027201178 | no | -1.354230447 | no |
| NGEG_RS0104945 | *traW* | -1.787387926 | yes | -1.861329498 | no |
| NGEG_RS0104950 | *traU* | -2.012735204 | no | -1.834226021 | no |
| NGEG_RS0104955 | *trbC* | -1.865348428 | no | -1.600824144 | no |
| NGEG_RS0104960 | *ybi* | -1.787878641 | no | -1.645662553 | no |
| NGEG_RS0104965 | *traN* | -2.252005088 | yes | -1.936178768 | no |
| NGEG_RS0104970 | *ycb* | -2.39471225 | no | -1.753854539 | no |
| NGEG_RS0104975 | *traF* | -2.297997944 | yes | -1.68036631 | no |
| NGEG_RS0104980 | *traH* | 2.478091465 | yes | 1.748400729 | yes |
| NGEG_RS0104985 | *traG* | -1.093409239 | no | -1.328919861 | no |
| NGEG_RS0104990 | *atlA* | -2.662624591 | yes | -1.77586658 | yes |
| NGEG_RS0104995 | *exp1* | -1.868215212 | yes | -1.112083627 | no |
| NGEG_RS0105000 | *cspA* | -1.438800881 | yes | -1.368217658 | no |
| NGEG_RS0105005 | *exp2* | -1.338987373 | no | -1.110407895 | no |
| NGEG_RS0105010 | *yda* | 1.03793262 | no | -1.032841316 | No |
| NGEG_RS0105015 | *ydbA* | -1.058236659 | no | 1.297303842 | No |
| NGEG_RS0105020 | *ydbB* | -1.80477177 | yes | -2.112006902 | Yes |
| NGEG_RS0105025 | *ydcA* | -1.949081316 | yes | -2.417834252 | Yes |
| NGEG_RS0105035 | *ydcB* | -2.305645862 | yes | -1.10353795 | No |
| NGEG_RS0105040 | *ydd* | -1.288012845 | no | -1.469797329 | No |
| NGEG_RS0105045 | *ydeA* | -1.092540243 | no | -1.243746693 | No |
| NGEG_RS0105050 | *ydeB* | -1.410996946 | no | 1.341547303 | No |
| NGEG_RS0105055 | *ydf* | -1.707746182 | yes | -1.151356411 | No |
| NGEG_RS14465 | *ydg* | -2.805134867 | yes | -1.23695979 | No |
| NGEG_RS0105065 | *ydhA* | -1.752581058 | yes | -1.38773991 | No |
| NGEG_RS0105070 | *ydhB* | 1.546256139 | yes | 1.450627141 | No |
| NGEG_RS0105075 | *ydi* | 1.112316881 | no | -1.012392396 | No |
| NGEG_RS0105080 | *yea* | 1.059333017 | no | -1.082448548 | No |
| NGEG_RS0105085 | *yeb* | -1.39331491 | no | -1.136446108 | No |
| NGEG_RS0105090 | *yecA* | -1.084193104 | no | -1.488501851 | No |
| NGEG_RS0105095 | *yecB* | -1.226044815 | no | -1.790755018 | No |
| NGEG_RS0105100 | *yedA* | -1.186287417 | no | -1.504557089 | No |
| NGEG_RS0105105 | *yedB* | -2.113912004 | yes | -2.407236543 | Yes |
| NGEG_RS14470 | *yee* | -1.239173395 | no | -2.066699364 | Yes |
| NGEG_RS0105110 | *NEIS2314* | 1.809390661 | no | -1.340026577 | No |
| NGEG_RS0105115 | *NEIS2313* | -1.264627136 | no | -1.761818858 | Yes |
| NGEG_RS0105120 | *yegA* | -1.622011225 | no | -1.144037569 | No |
| NGEG_RS0105125 | *yeh* | -1.139090743 | no | -1.049849602 | No |
| NGEG_RS0105130 | *topB* | 1.113716959 | no | 1.093663407 | No |
| NGEG_RS14475 |  | 1.092131536 | no | 1.334977207 | No |
| NGEG_RS14480 | *ssb* | 1.398926973 | no | 1.552722311 | No |
| NGEG_RS0105140 | *yfa* | -1.66401128 | yes | -1.377155726 | No |
| NGEG_RS0105145 | *yfb* | -2.452355298 | yes | -1.978427714 | Yes |
| NGEG_RS12710 | *yfd* | -1.811329151 | no | -1.54065503 | No |
| NGEG_RS0105155 | *yfeA* | -1.248912412 | no | -1.096740881 | No |
| NGEG_RS0105160 | *yfeB* | -1.381992536 | no | -1.673012874 | No |
| NGEG_RS0105165 | *parB* | -1.661490671 | no | -1.857705466 | No |
| NGEG_RS0105170 | *parA* | -1.241122972 | no | -1.44447283 | No |

^a^ Genes highlighted in red were differentially regulated in the indicated regulon at ≥2-fold change and achieving statistical significance. The gene annotation was adopted after the nomenclature used for the MS11 strain GGI reported by Ramsey et al. [[70](#_ENREF_70)]. The specific gene sizes and positions in FA19 GGI vary slightly from the MS11 GGI annotation.
