# Supplementary material for: Transcriptional responses of Neisseria gonorrhoeae to glucose and lactate: implications for resistance to oxidative damage and biofilm formation
Source: mBio. 2024 Jul 16;15(8):e01761-24. doi: 10.1128/mbio.01761-24 (PMC11323468; doi:10.1128/mbio.01761-24)
Supplement: Table S6 — Oligonucleotide primers used in this study. [file mbio.01761-24-s0010.docx]

**Table S6. Oligonucleotide primers used in this study.**

| **Forward primer** | Forward primer sequence (5′ **to 3′)** | Reverse primer | Reverse primer sequence (5′ **to 3′)** | **Name gene target** | **NGO tag number** | **Protein name /cellular function** |
| --- | --- | --- | --- | --- | --- | --- |
| recAqFw | AACCTCGAAGTCATTTCCACCGG | recAqRv | TCTGGCATTGGGCGACGGCTTC | *recA* | NGO0741 | Recombinase A |
| 16Smai-RTF | CCATCGGTATTCCTCCACATCTCT | 16Smai-RTR | CGTAGGGTGCGAGCGTTAATC | *16S rRNA* | NGO_18906 | 16S rRNA |
| lctPqFw | CGCCATCAAACTTTTCTACTTCGG | lctPqRv | ACATCGATGCAGCCCGTGGTTTC | *lctP* | NGO1449 | L-lactate permease |
| pilinFw | GGGTTTACGCTGGTTGAATTAATAT | pilinRv | CATTTGGTAGATGTTTGTTTAAATCT | *Pilin* | NGO1177 | Neisseria-specific type VI pilin-related protein |
| pykAqFw | CCACAACACCAAAATCGTCGCC | pykAqRv | GTTTTGCCGCCTCGCGCACGA | *pykA* | NGO1881 | pyruvate kinase |
| G6PDqFw | TTTGGTGTTGTTCGGTGCGACC | G6PDqRv | TTTCACATGGATTTTGGAACTGGTTT | *G6PD* | NGO0715 | glucose-6-phosphate dehydrogenase |
| sucDqFw | TAAAGTATTGGTTCAAGGTTTCACCG | sucDqRv | CAGGCAGGTCTAGGTGGGTTTG | *sucD* | NGO0912 | succinate-CoA ligase subunit alpha |
| hexRqFw | AGCAAAATCAGCGAATCACTGGCC | hexRqRv | GAGAGCTTGAATTCCGGCAGCC | *hexR* | NGO0718 | glucose catabolism regulator |
| ppkqFw | GTTTGAAAACGTAGAATTAGGCGAAA | ppkqRv | CATACGTTTGCGGCAGGGGTAGT | *Ppk* | NGO2113 | polyphosphate kinase 2 |
| gapDHqFw | CCGCATCGGCCGCCTCGCATT | gapDHqRv | GCCTTGTGTGCTGTCGTATTTGAAA | *gapDH* | NGO1931 | type I glyceraldehyde-3-phosphate dehydrogenase |
| nqrBqFw | GAAAAAATCGAACCGCACTTCCTG | nqrBqRv | AACAAAGCCAGCCACACCAAAATC | *Nqr* | NGO1414 | NADH:ubiquinone reductase (Na(+)-transporting) subunit B |
| atpDqFw | TCGGTGCGGTTGTTGACGTGGAA | atpDqRv | ACAGTAATGGGCGAACCAGTATTG | *atpD* | NGO2150 | ATP synthase subunit beta |
| mtrR_qRT_F | CTTGTTTGACGCGTTGTTCCA | mtrR_qRT_R | GTGGATGTCGTTGCTTTGCA | *mtrR* | NGO1366 | MtrCDE efflux pump gene repressor |
| katAqRT-F | CCATCTGACCATGAACAACGGCG | katAqRT-R | GGTAAACGTACCGAACGCGCCC | *katA* | NGO1767 | H_2_O_2_-inducible catalase |
| exbBqFw | AGCTTGGATGTTTGATTGGTTTTTTC | exbBRv | GCAAGACAACCAGACACCACGTT | *exbB* | NGO1378 | inner membrane protein that interacts with TonB |
| tonBqFw | TTTTAACCCCCGCAGTCGTGTTTT | tonBqRv | CGCCGCCCCCTCCGCCAAAA | *tonB* | NGO1379 | inner membrane protein that interacts with high affinity outer membrane receptors and participate in transport processes |
| tbpA_qRT_F | TTTCGACACGCGCGATATGA | tbpA_qRT_R | AGTCCGCCGTATTTGTGGTT | *tbpA* | NGO1495 | transferring-binding protein A |
| fbpCqFw | CCCTGACCGCCGCCACCCC | fbpCqRv | CACTGTTGAGTTTGACTTTGATGCC | *fbpC* | NGO0217 | Iron ABC trnasporter substrate binding protein |
| fetA_qRT_F | AGAGTTTGCCGTCAGCGAAA | fetA_qRT_R | TAGGCGTTGGCATCCAGTTT | *fetA* | NGO2093 | TonB-dependent siderophore receptor |
| mafAqFw | CTGCTCCTCCTCATCCCCCTC | mafAqRv | TCCGCCGCTTATGTTGCCCGAA | *mafA* | NGO1393 and NGO1584 | Adhesin MafA |
| lrpqFw | ATTTCCGCATCCTCAAAATTCTTCA | lrpqRv | AATATAATGTTCCCGCTCCAAACG | *lrp* | NGO1294 | Lrp/AsnC family transcriptional regulator |
| mntCqFw | GCCACCGCCGCAACTGCCGC | mntCqRv | AAGTCCCAAGCCGTTGAGCAGGA | *mntC/znuA* | NGO0168 | periplasmic Zn^2+^-binding protein associated with the high-affinity ATP-binding cassette ZnuABC |
